# Identification of Risk Factors for Chronic Q Fever, the Netherlands

**DOI:** 10.3201/eid1804.111478

**Published:** 2012-04

**Authors:** Linda M. Kampschreur, Sandra Dekker, Julia C.J.P. Hagenaars, Peter J. Lestrade, Nicole H.M. Renders, Monique G.L. de Jager-Leclercq, Mirjam H.A. Hermans, Cornelis A.R. Groot, Rolf H.H. Groenwold, Andy I.M. Hoepelman, Peter C. Wever, Jan Jelrik Oosterheert

**Affiliations:** University Medical Centre Utrecht, Utrecht, the Netherlands (L.M. Kampschreur, R.H.H. Groenwold, A.I.M Hoepelman, J.J. Oosterheert);; VU University, Amsterdam, the Netherlands (S. Dekker);; Jeroen Bosch Hospital, ’s-Hertogenbosch, the Netherlands (J.C.J.P. Hagenaars, P.J. Lestrade, N.H.M. Renders, M.H.A. Hermans, P.C. Wever);; Bernhoven Hospital, Oss/Veghel, the Netherlands (M.G.L. de Jager-Leclercq, C.A.R. Groot)

**Keywords:** Q fever, Coxiella burnetii, chronic Q fever, risk factors, case–control study, acute Q fever, cardiac valvular surgery, vascular prosthesis, aortic aneurysm, renal insufficiency, older age, bacteria, the Netherlands, *Suggested citation for this article*: Kampschreur LM, Dekker S, Hagenaars JCJP, Lestrade PJ, Renders NHM, de Jager-Leclercq MGL, et al. Identification of risk factors for chronic Q fever, the Netherlands. Emerg Infect Dis [serial on the internet]. 2012 Apr [*date cited*]. http://dx.doi.org/10.3201/eid1804.111478

## Abstract

Since 2007, the Netherlands has experienced a large Q fever outbreak. To identify and quantify risk factors for development of chronic Q fever after *Coxiella burnetii* infection, we performed a case–control study. Comorbidity, cardiovascular risk factors, medications, and demographic characteristics from 105 patients with proven (n = 44), probable (n = 28), or possible (n = 33) chronic Q fever were compared with 201 patients who had acute Q fever in 2009 but in whom chronic Q fever did not develop (controls). Independent risk factors for development of proven chronic Q fever were valvular surgery, vascular prosthesis, aneurysm, renal insufficiency, and older age.

## Abstract 2

Previous cardiac valvular surgery, vascular prosthesis, aortic aneurysm, renal insufficiency, and older age increased risk.

Q fever, a zoonosis caused by the intracellular gram-negative bacterium *Coxiella burnetii*, is prevalent worldwide (*1**,**2*) and has various acute and chronic clinical manifestations. Acute Q fever is mostly a self-limiting, mild, influenza-like disease, sometimes complicated by severe pneumonia or hepatitis. Asymptomatic acute infection occurs in 50%–60% of patients (*3**–**5*). Among patients infected by *C. burnetii*, infection progresses to chronic Q fever in 1%–5%, months to years after primary infection (*2**,**4**,**6*). Previous data, mainly from France, show that endocarditis is the most common clinical manifestation (±75%), followed by infections of aortic aneurysms and vascular prostheses (±10%) (*5**,**7**–**9*). In the Netherlands, however, an equal distribution of endocarditis and vascular infections has been seen (*10*).

Chronic Q fever leads to high illness and death rates if untreated, which makes early case finding and preventive measures critical for patients at high risk. Treatment for Q fever consists of long-term antimicrobial drug therapy, preferably a combination of doxycycline and hydroxychloroquine for 18–24 months. Previously identified risk factors for chronic Q fever are preexisting cardiac valvulopathy, vascular grafts and aneurysms, immunosuppression, and pregnancy; however, most published studies have been descriptive, lacked statistical quantification, or included specific high-risk groups only (*6**–**9**,**11**,**12*).

Since 2007, a large Q fever outbreak has been ongoing in the Netherlands, with >4,000 acute Q fever cases reported (*13*). Because of asymptomatic disease and overlap with other febrile diseases, however, the actual number of Q fever infections is probably much higher. Although the acute Q fever epidemic in the Netherlands has subsided, the number of patients with chronic Q fever is rising (*10**,**14*). In this unique population, we conducted a case–control study to identify and quantify risk factors for development of chronic Q fever after *C. burnetii* infection.

## Methods

### Study Design and Setting

Case-patients and controls were recruited from Jeroen Bosch Hospital in ’s-Hertogenbosch and Bernhoven Hospital in Oss and Veghel; both are regional hospitals located in the center of the Q fever epidemic area in the Netherlands. The study design was approved by the Medical Research Ethics Committee of the University Medical Centre Utrecht.

### Patient Selection

We used existing datasets and spontaneous notifications from the 2 hospitals to identify all chronic Q fever diagnoses among patients >18 years of age during January 1, 2007–May 1, 2011. In the past, diagnosis of chronic Q fever has relied on results of serologic testing and PCR. Chronic Q fever is considered proven if *C. burnetii* is detected by PCR in blood or tissue in the absence of acute infection, but sensitivity of this technique is only ≈50% (*15**,**16*). Persisting high levels of IgG to phase I antigens (phase I IgG) and, to a lesser extent, phase II antigens (phase II IgG) are also indicative of chronic Q fever (*1*). The optimal immunofluorescence assay (IFA) cutoff value for phase I IgG titer is still matter of debate and is dependent on the test used but is probably within the range of 800–1,600 (*7**,**17**–**19*).

Recently, the Dutch Q Fever Consensus Group proposed a new diagnostic approach that combines PCR, serologic testing, and clinical data and categorizes cases into proven, probable, or possible chronic Q fever (*20*). Proven cases are those among patients with positive PCR results for *C. burnetii* in blood or tissue or a phase I IgG titer of >1,024 in combination with a vascular infection proven by positron emission tomography (PET), computed tomography (CT), magnetic resonance imaging (MRI), or endocardial involvement according to the major criteria of the modified Duke criteria on echocardiogram (*21*). Probable cases are those among patients with phase I IgG titers of >1,024 and known risk factors: nonmajor valvulopathy according to the modified Duke criteria (*21*), suspected nonvascular or noncardial localization of chronic Q fever infection, or aspecific signs of chronic infection. Possible cases are those among patients with phase I IgG titers of >1,024 without other risk factors as listed for probable or proven chronic Q fever. In contrast to the other 2 subgroups, in general, possible chronic Q fever patients do not receive long-term antimicrobial drug treatment but instead enter a follow-up program; many demonstrate spontaneous decline in phase I IgG titers. We defined cases according to these definitions (*20*) (Table 1).

**Table 1:** Classification of chronic Q fever according to Dutch Q Fever Consensus Group guidelines*

Classification	Definition
Proven	Any of the following:
	Positive PCR for *Coxiella burnetii* in serum, plasma, or tissue in the absence of acute Q fever
	IFA phase I titer ≥1,024 with definite endocarditis according to the revised Duke criteria (*21*)
	Indication of vascular infection on PET/CT, CT, MRI, or ultrasound testing
Probable	IFA phase I IgG titer >1,024 and any of the following clinical manifestations:
	Valvulopathy not meeting the criteria of endocardial involvement of the major modified Duke criteria (*22*)
	Aneurysm, vascular prosthesis or prosthetic valve without signs of infection on PET/CT, CT, MRI, or ultrasound testing
	Signs of possible chronic Q fever infection of noncardiac or vascular origin on PET/CT, CT, or ultrasound testing
	Pregnancy
	Clinical symptoms of chronic infection (i.e., fever, night sweats, weight loss, hepatosplenomegaly)
	Histopathologic proven granulomatous inflammation
	Immune disorder
Possible	IFA phase I IgG titer >1,024 without clinical manifestations as described above

*Described in (*20*). IFA, immunofluorescence assay; PET, positron emission tomography; CT, computed tomography; MRI, magnetic resonance imaging.

Controls were selected from an existing cohort of patients with acute Q fever, seen by general practitioners in 2009, who had positive PCR results for *C. burnetii* in serum samples. Controls were included if they were >18 years of age at the time of acute Q fever and if the serologic profile was not suggestive of chronic Q fever during >1 year of follow-up (i.e., decreasing antibody titers and phase I IgG titer <1,024). Patients with serologic follow-up of <1 year after the episode of acute Q fever were excluded from analysis. All case-patients except 1 and all controls lived in the same postal code area (5000–5400) in the Netherlands.

### Microbiological Analyses

Microbiological diagnostics for chronic Q fever case-patients consisted of IFA (Focus Diagnostics, Inc., Cypress, CA, USA) of serum samples and PCR for *C. burnetii* DNA in serum, plasma, and tissue samples. The diagnostic workup to evaluate *C. burnetii* infection in control patients with documented acute Q fever had been performed according to a diagnostic algorithm for acute Q fever introduced in May 2009. In brief, serum samples were screened with ELISA for IgM against *C. burnetii* phase II antigens (MII-screen; Institut Virion Serion GmbH, Würzburg, Germany). Depending on date of onset of disease and inpatient or outpatient setting, PCR for *C. burnetii* DNA was performed if the MII-screen result was negative (*23**–**25*). In patients with confirmed acute Q fever, serologic follow-up was performed at 3, 6, and 12 months, consisting of IFA for IgM and IgG against *C. burnetii* phase I and phase II antigens.

### Data Collection and Storage

We collected patient characteristics including demographic variables, medical history, medication, pathology and microbiology results, imaging records, therapy, and outcome for case-patients and controls. Case-patient information was already available in the hospital registration systems and was interpreted by 2 researchers (L.K. and S.D.). All controls were sent a questionnaire and an informed consent form that asked for permission to request patient’s data from the general practitioner and from the hospital registration system.

Although debatable, routine echocardiographic screening after diagnosis of acute Q fever is not the standard of care in the Netherlands because no benefit was found in an earlier evaluation (*26**,**27*). Therefore, for chronic Q fever case-patients and acute Q fever controls, details about cardiac valvulopathy were retrieved by review of medical records. The obtained information was processed and stored anonymously with the use of coded data. SPSS version 18.0 was used for storage and analysis of the collected data (SPSS Inc., Chicago, IL, USA).

### Statistical Analysis

Within this study, we conducted 3 analyses: 1) an overall analysis of all chronic Q fever cases (i.e., proven, probable, and possible); 2) an analysis of proven and probable chronic Q fever cases; and 3) an analysis of proven chronic Q fever cases only. We performed these analyses to determine whether exclusion of possible chronic Q fever and, to a lesser extent, probable chronic Q fever (the groups in which disease status is doubtful) influenced the overall results. Univariate and subsequent multivariate logistic regression analyses were performed to calculate odds ratios (ORs), corresponding 95% CIs, and p values for the development of chronic Q fever. In univariate analysis, missing values were excluded. Variables with no observations among case-patients and <2 observations in the control group (or vice versa) were excluded (i.e., hematologic malignancies, bone marrow transplantation, dialysis, renal transplant, nonrenal organ transplant, congenital cardiac deviation, pulmonary diseases, and autoimmune disorder). For potential dichotomous risk factors, i.e., those that had 0 observations among either the case-patients or controls but >2 observations in the other, we applied a Fisher exact test to calculate p values. Variables with >1 observations and <25% missing values in case-patients and controls, a p value of <0.10 in univariate analysis, or known association in previous reports with the development of chronic Q fever were subsequently analyzed in a multivariate model.

The variables vascular history and valvulopathy were not included in multivariate analysis because they were included in variables that were listed separately (i.e., vascular prosthesis, aneurysm, other vascular surgery, peripheral arterial disease, cerebrovascular disease, valvular surgery, and nonsurgical valvular disease). Eighteen case-patients and 0 controls had a history of valvular surgery. Because of the expected importance of this risk factor and the high incidence among case-patients, we considered its inclusion in the multivariate analysis critical; moreover, the logistic regression model could not be fitted with this variable excluded. Therefore, we randomly changed one of the observations of the control group from 0 to 1, which artificially reduced the association but enabled us to fit the regression model.

The variables age, vascular history, vascular prosthesis, aneurysm, other vascular surgery, cerebrovascular disease, peripheral vascular disease, valvulopathy, valvular surgery, valvular deviation, ischemic heart disease, other cardiovascular diseases, hypertension, dyslipidemia, diabetes, nonhematologic malignancy, and renal insufficiency could be included in multivariate analysis of all groups. The variable immune disorder was also included in multivariate analysis for the probable and proven and the proven subgroups. The variable pacemaker was also included in multivariate analysis for the proven group.

Differences between case-patients and controls were shown in use of statins, clopidogrel, acenocoumarol, and proton pump inhibitors and hospitalization and adequate treatment during acute Q fever (p<0.10). However, these variables could not be included in the multivariate analysis because >25% of values were missing, most among case-patients in whom an acute Q fever episode had gone unrecognized.

After selecting predictors for our final multivariate model, we evaluated their possible interactions by including 2-way interactions in consecutive models. Interactions were not significant and therefore not included in the model. To assess the goodness-of-fit of the final model, we plotted sensitivity and specificity by using a receiver operating characteristic curve and estimated the area under the curve (c-statistic). p<0.05 was considered significant.

## Results

We identified 105 case-patients with proven, probable, or possible chronic Q fever; 44 (42%) had proven, 28 (27%) probable, and 33 (31%) possible disease. Of the case-patients with proven chronic Q fever, 27 (61%) had positive PCR results for *C. burnetii* in blood only, 5 (11%) in tissue only, 8 (18%) in tissue and blood, and 4 (9%) in neither blood nor tissue. The focus of infection in cases of proven chronic Q fever was endocarditis for 12 case-patients (27%) and endovascular infection for 26 (59%); 6 (14%) had no clear infection focus. Of the case-patients with probable chronic Q fever, suspected foci were cardiac valves in 12 (43%), endovascular lesions in 1 (4%), and another focus (e.g., pregnancy or clinical symptoms of infection such as weight loss, night sweats, and fever) in 15 (54%).

Long-term antimicrobial drug treatment was started for 40/44 case-patients (91%) with proven chronic Q fever, 18/28 case-patients (64%) with probable chronic Q fever, and 5/32 case-patients (15%) with possible chronic Q fever. Three patients with proven chronic Q fever patients died before diagnosis of chronic Q fever; 1 refused therapy.

**Figure 1:**
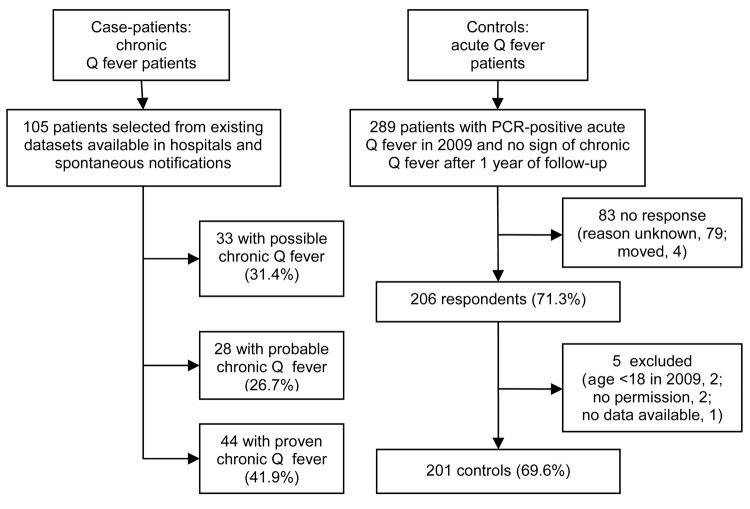
Enrollment, selection, and inclusion criteria forcase-patients and controls for case–control study to identify risk factors for chronic Q fever, the Netherlands.

In all, 289 controls who had PCR-proven acute Q fever in 2009 were sent a questionnaire. Of these, 201 (69.6%) responded, signed the informed consent form, and fulfilled the inclusion criteria (Figure 1).

Results of the univariate analysis are listed in Table 2. Comparisons for age, vascular history, vascular prosthesis, aneurysm, other vascular surgery, cerebrovascular disease, peripheral vascular disease, valvulopathy, valvular surgery, valvular deviation, ischemic heart disease, other cardiovascular diseases, hypertension, dyslipidemia, diabetes, nonhematologic malignancy (defined as several kinds of solid tumors), renal insufficiency, and pregnancy showed significant differences between case-patients and controls.

**Table 2:** Results of univariate analysis of risk factors for chronic versus acute Q fever*

Risk factor	Acute Q fever, no. (%), n = 201	All chronic Q fever, n = 105				Proven and probable chronic Q fever, n = 72				Proven chronic Q fever, n = 44		
		No. (%)	OR (95% CI)	p value		No. (%)	OR (95% CI)	p value		No. (%)	OR (95% CI)	p value
Male	129 (64.2)	70 (66.7)	1.12 (0.68–1.84)	0.665		50 (69.4)	1.27 (0.71–2.26)	0.420		32 (72.7)	1.49 (0.72–3.07)	0.281
Mean age, y (±SD)	52.5 (±13.7)	63.9 (±13.5)	1.06 (1.04–1.09)†	0.000		67.3 (±11.8)	1.09 (1.07–1.12)†	0.000		68.4 (±10.8)	1.11 (1.07–1.15)†	0.000
Smoker	85 (42.5)	43 (44.3)	1.08 (0.66–1.76)	0.765		33 (49.3)	1.31 (0.75–2.29)	0.336		22 (55.0)	1.65 (0.84–3.27)	0.149
Medical history												
Vascular history	9 (4.5)	33 (31.4)	9.78 (4.46–21.4)	0.000		29 (40.3)	14.4 (6.35–32.6)	0.000		23 (52.3)	23.4 (9.57–57.1)	0.000
Vascular prosthesis	2 (1.0)	15 (14.3)	16.5 (3.71–74.0)	0.000		15 (20.8)	26.2 (5.82–118)	0.000		14 (31.8)	46.4 (10.0–215)	0.000
Aneurysm	2 (1.0)	12 (11.4)	12.8 (2.82–58.5)	0.001		12 (16.7)	19.9 (4.33–91.4)	0.000		9 (20.5)	25.6 (5.30–123)	0.000
Other vascular surgery	3 (1.5)	7 (6.7)	4.71 (1.19–18.6)	0.027		5 (6.9)	4.93 (1.15–21.2)	0.032		4 (9.1)	6.60 (1.42–30.6)	0.016
Peripheral arterial disease	6 (3.0)	11 (10.5)	3.80 (1.37–10.6)	0.011		8 (11.1)	4.06 (1.36–12.2)	0.012		6 (13.6)	5.13 (1.57–16.8)	0.007
Cerebrovascular disease‡	8 (4.0)	11 (10.5)	2.82 (1.10–7.25)	0.031		9 (12.5)	3.45 (1.28–9.31)	0.015		5 (11.4)	3.09 (0.96–9.96)	0.058
Valvulopathy	10 (5.0)	25 (23.8)	5.97 (2.47–13.0)	0.000		23 (31.9)	8.97 (4.00–20.1)	0.000		13 (29.5)	8.01 (3.23–19.8)	0.000
Valvular disease, NS§	10 (5.0)	17 (16.2)	3.69 (1.62–8.39)	0.002		14 (19.4)	4.61 (1.95–10.9)	0.001		9 (20.5)	4.91 (1.86–13.0)	0.001
Valvular surgery	1 (0.5)¶	18 (17.1)	41.4 (5.44–315)	0.000		18 (25.0)	66.7 (8.70–511)	0.000		10 (22.7)	58.8 (7.29–474)	0.000
Congenital cardiac disease	1 (0.5)	1 (1.0)	1.92 (0.12–31.1)	0.645		NA	NA	NA		NA	NA	NA
Ischemic cardiac disease#	17 (8.5)	28 (26.7)	3.94 (2.04–7.61)	0.000		23 (31.9)	5.08 (2.52–10.2)	0.000		17 (38.6)	6.82 (3.11–14.9)	0.000
Pacemaker	2 (1.0)	3 (2.9)	2.93 (0.48–17.8)	0.244		3 (4.2)	4.33 (0.71–26.4)	0.113		3 (6.8)	7.28 (1.18–45.0)	0.033
Other cardiac history**	12 (6.0)	26 (24.8)	5.18 (2.49–10.8)	0.000		23 (31.9)	7.39 (3.44–15.9)	0.000		15 (34.1)	8.15 (3.47–19.1)	0.000
Hypertension	56 (27.9)	44 (41.9)	1.87 (1.14–3.07)	0.013		35 (48.6)	2.45 (1.41–4.27)	0.002		24 (54.5)	3.11 (1.59–6.06)	0.001
Dyslipidemia	39 (19.4)	32 (30.5)	1.82 (1.06–3.13)	0.031		23 (31.9)	1.95 (1.06–3.58)	0.031		16 (36.4)	2.37 (1.17–4.81)	0.017
Diabetes mellitus, type 1 or 2	13 (6.5)	15 (14.3)	2.41 (1.10–5.28)	0.028		10 (13.9)	2.33 (0.97–5.58)	0.057		7 (15.9)	2.74 (1.02–7.32)	0.045
Nonhematologic malignancy	6 (3.0)	16 (15.2)	5.84 (2.21–15.4)	0.000		10 (13.9)	5.24 (1.83–15.0)	0.002		6 (13.6)	5.13 (1.57–16.8)	0.007
Immune disorder††	2 (1.0)	4 (3.8)	3.94 (0.71–21.9)	0.117		4 (5.6)	5.85 (1.05–32.7)	0.044		3 (6.8)	7.28 (1.18–45.0)	0.033
COPD	14 (7.0)	13 (12.4)	1.89 (0.85–4.18)	0.117		9 (12.5)	1.91 (0.79–4.62)	0.152		6 (13.6)	2.11 (0.76–5.84)	0.151
Other pulmonary disease‡‡	6 (3.0)	3 (2.9)	0.96 (0.23–3.90)	0.950		2 (2.8)	0.93 (0.18–4.71)	0.929		NA	NA	NA
Liver disease	1 (0.5)	3 (2.9)	5.88 (0.60–57.3)	0.127		2 (2.8)	5.71 (0.51–64.0)	0.157		1 (2.3)	4.65 (0.29–75.8)	0.280
Renal insufficiency	2 (1.0)	12 (11.4)	12.8 (2.82–58.5)	0.001		12 (16.7)	19.9 (4.33–91.4)	0.000		9 (20.5)	25.6 (5.30–123)	0.000
Autoimmune disease§§	2 (1.0)	1 (1.0)	0.96 (0.09–10.7)	0.971		1 (1.4)	1.40 (0.13–15.7)	0.784		NA	NA	NA
Pregnancy¶¶	0 (0.0)	3 (2.9)	NA	0.040		2 (2.8)	NA	0.069		1 (2.3)	NA	0.180
Medication at time of acute Q fever												
Proton pump inhibitors¶¶	15 (7.5)	7 (11.7)	1.63 (0.63–4.20)	0.313		5 (14.7)	2.13 (0.72–6.29)	0.173		5 (23.8)	3.85 (1.24–12.0)	0.020
Statin¶¶	29 (14.5)	19 (31.7)	2.73 (1.40–5.35)	0.003		15 (44.1)	4.66 (2.13–10.2)	0.000		13 (61.9)	9.58 (3.65–25.1)	0.000
Carbasalate calcium¶¶	6 (3.0)	2 (3.3)	1.12 (0.22–5.67)	0.896		2 (5.9)	2.02 (0.39–10.5)	0.401		2 (9.5)	3.40 (0.64–18.0)	0.150
Acenocoumarol¶¶	6 (3.0)	7 (11.7)	4.27 (1.38–13.3)	0.012		5 (14.7)	5.58 (1.60–19.4)	0.007		2 (9.5)	3.40 (0.64–18.0)	0.150
Clopidogrel¶¶	2 (1.0)	3 (5.0)	5.21 (0.85–31.9)	0.074		2 (5.9)	6.19 (0.84–45.5)	0.073		2 (9.5)	10.4 (1.39–78.2)	0.023
Acute Q fever												
Adequate treatment##	157 (89.7)	37 (84.1)	0.61 (0.24–1.56)	0.298		22 (78.6)	0.42 (0.15–1.17)	0.098		12 (70.6)	0.28 (0.09–0.87)	0.028
Hospitalization	36 (18.0)	26 (35.1)	2.47 (1.36–4.49)	0.003		16 (38.1)	2.80 (1.37–5.76)	0.005		9 (34.6)	2.41 (0.99–5.84)	0.051

*No. (%) case patients. n indicates no. patients wth information available for that category. OR, odds ratio; NS, nonsurgical; NA, not applicable; COPD, chronic obstructive pulmonary disease. †OR per year of increasing age. ‡Cerebrovascular disease and transient ischemic attack. §Case-patients: aortic valve defects, 10 (no bicuspid valves); mitral valve defects, 9 (no prolapse); tricuspid valve defects, 4. Controls: aortic valve defects, 6 (no bicuspid valves); mitral valve defects, 3 (1 prolapse). ¶n = 0 in reality. #Angina pectoris and myocardial infarction. **Atrial fibrillation, congestive heart failure, pericarditis, bradycardia, ischemic cardiomyopathy, and left ventricular hypertrophy. ††Prednisone cumulative dose >750 mg; use of tumor necrosis factor α–blocker, methotrexate, mycofenolate mofetil; splenectomy. §§Asthma, recurrent pneumonia, rheumatoid arthritis. ¶¶>25% missing in case groups. ##Defined as 10–14 d of doxycycline treatment

**Figure 2:**
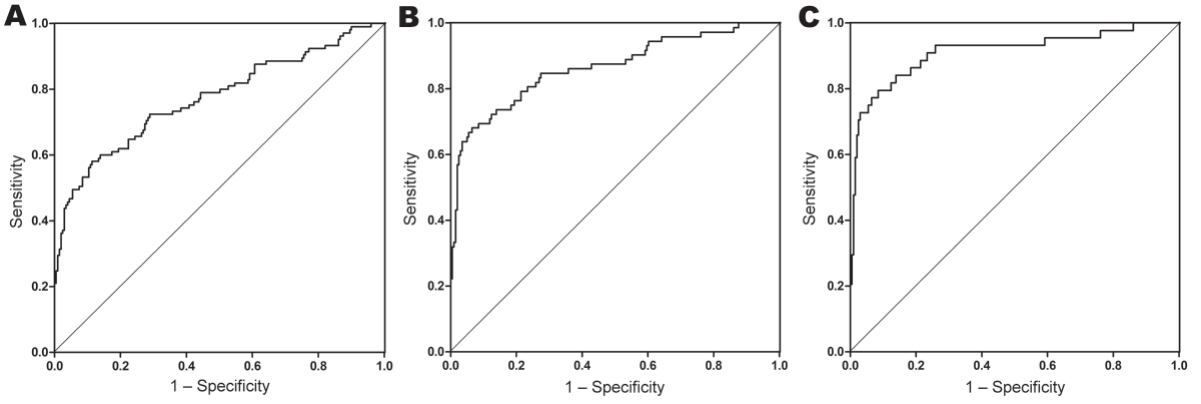
Goodness-of-fit models for case–control study to identify risk factors for chronic Q fever, the Netherlands. A) All chronic Q fever cases (n = 105); area under the curve (c-statistic) 0.77 (95% CI 0.71–0.83); p<0.001. B) Proven and probable chronic Q fever cases (n = 72); c-statistic 0.86 (95% CI 0.81–0.92); p<0.001. C) Proven chronic Q fever cases (n = 44); c-statistic 0.91 (95% CI 0.85–0.97); p<0.001. Patient risk factors included in the model (no. observations): A) valvular surgery (18); vascular prosthesis (15); aneurysm (12); nonhematologic malignancy (16); age, continuous, mean 63.9 y; B) valvular surgery (18); vascular prosthesis (15); aneurysm (12); renal insufficiency (12); age, continuous, mean 67.3 y; C) valvular surgery (10); vascular prosthesis (14); aneurysm (9); renal insufficiency (9); age, continuous, mean 68.4.

Results of the multivariate analyses are shown in Table 3. Valvular surgery (OR 31.5, 95% CI 3.99–249), vascular prosthesis (OR 10.4, 95% CI 2.17–50.0), aneurysm (OR 8.65, 95% CI 1.74–42.9), nonhematologic malignancy (OR 3.90, 95% CI 1.33–11.5), and age (OR 1.03, 95% CI 1.01–1.06) were independently associated with the development of chronic Q fever. The final discriminative performance was good, with a c-statistic of 0.71 (95% CI 0.71–0.83) (Figure 2).

**Table 3:** Results of multivariate analyses of risk factors for development of chronic Q fever, the Netherlands*

Risk factor*†	All chronic Q fever			Proven and probable chronic Q fever			Proven chronic Q fever	
	OR (95% CI)	p value		OR (95% CI)	p value		OR (95% CI)	p value
Valvular surgery†‡	31.5 (3.99–249)	0.001		47.7 (5.87–387)	0.000		43.6 (4.70–405)	0.001
Vascular prosthesis‡§	10.4 (2.17–50.0)	0.003		14.9 (2.96–75.2)	0.001		26.8 (4.88–147)	0.000
Aneurysm§¶	8.65 (1.74–42.9)	0.008		13.5 (2.60–70.4)	0.002		25.9 (4.55–147)	0.000
Renal insufficiency¶#	–	–		9.08 (1.44–57.2)	0.019		16.0 (2.06–123)	0.008
Nonhematologic malignancy	3.90 (1.33–11.5)	0.013		–	–		–	–
Age, continuous	1.03 (1.01–1.06)#	0.005		1.06 (1.03–1.09)#	0.000		1.06 (1.02–1.11)#	0.005

*OR, odds ratio. †Possible risk factors entered in all analyses: age, vascular prosthesis, aortic aneurysm, other vascular surgeries, peripheral arterial disease, cerebrovascular disease, valvular surgery, valvular disease (nonsurgical), ischemic cardiac disease, other cardiac history, hypertension, dyslipidemia, diabetes, nonhematologic malignancy, renal insufficiency. Immune disorder was also entered in the analyses of proven and probable chronic Q fever and of proven chronic Q fever. Pacemaker was also entered in the analysis of proven chronic Q fever. ‡Valvular surgeries in the proven group are subdivided into biological valve (n = 6), prosthetic valve (n = 3), and valve repair (n = 1) all located in the aortic valve (n = 10). Within the controls there were no patients with history of valvular surgery. §Locations of vascular prostheses in proven group were infrarenal and iliac (n = 6), infrarenal (n = 4), thoracic (n = 2), and unknown (n = 2). Types of vascular prosthesis were Y-prosthesis (n = 7), endovascular aneurysm repair (n = 2), stent graft (n = 2), Bentall (n = 1), and unknown (n = 2). For the 2 control patients, specifications of the prostheses were unknown. ¶Locations of aneurysms in proven group were infrarenal (n = 6), infrarenal and iliac (n = 2), and suprarenal, infrarenal, and iliac (n = 1). Within the control group, aneurysms were infrarenal and iliac (n = 2). #Observed stages of chronic kidney disease according to the Kidney Disease Outcome Quality Initiative guidelines (*28*) in the proven group were stage 3 (n = 6), stage 4 (n = 2), and stage 5 (n = 1) and in the controls solely stage 3 (n = 2). #OR per year of increasing age.

Patient risk factors identified in the analysis of the proven cases, representing the most definite chronic Q fever cases, were valvular surgery (OR 43.6, 95% CI 4.70–405), vascular prosthesis (OR 26.8, 95% CI 4.88–147), aneurysm (OR 25.9, 95% CI 4.55–147), renal insufficiency (OR 16.0, 95% CI 2.06–123), and age (OR 1.06, 95% CI 1.02–1.11). The final discriminative performance was good, with a c-statistic of 0.91 (95% CI 0.85–0.97) (Figure 2).

## Discussion

To our knowledge, this is the first study that analyzed a large number of potential risk factors for chronic Q fever in a large number of patients. Most former studies have been limited by a low number of cases and evaluation of few risk factors. Moreover, quantification of these risk factors was lacking (*6**–**9**,**11**,**12*).

In our study, we focused mainly on case-patients with proven chronic Q fever becausethis group included patients with the most definite form of chronic Q fever. Proven chronic Q fever also showed the strongest correlation with the identified risk factors. In multivariate analysis, valvular surgery, vascular prosthesis, aneurysms, renal insufficiency, and age were significant risk factors for the development of chronic Q fever in patients with proven cases. In the analysis of all patients with chronic Q fever cases, nonhematologic malignancy also seemed to be a risk factor; however, this could not be reproduced in the subanalyses of the more definite cases (e.g., proven and probable cases). Hence, nonhematologic malignancy as a risk factor remains uncertain. Valvular surgery, vascular prostheses, and aneurysms were the strongest predictors in this study, which confirms observational findings from earlier studies. Explanation lies in the association with the preferred localization of chronic Q fever infection.

A novel finding is the association between mild renal insufficiency and chronic Q fever. The majority of patients with chronic Q fever and renal disease in our study had stage 3 renal insufficiency according to Kidney Disease Outcome Quality Initiative guidelines (*28*). Although terminal renal insufficiency can decrease the immune response, this association was not found for mild renal disease (*29*). Renal insufficiency is associated with vascular disease, which may explain the elevated incidence of chronic Q fever in these patients (*30*).

Increasing age also predisposes for the development of chronic Q fever; this predisposition was also illustrated in a recent report of van der Hoek et al. (*24*). The explanation probably lies in the increased prevalence of cardiovascular diseases and the decreased cellular immunity during aging (*31**,**32*). Age >60 years appeared the best cutoff above which the risk for chronic Q fever increases significantly.

Preexisting cardiac valvulopathy has been found to give an estimated risk of 39% for the development of chronic Q fever after infection with *C. burnetii* (*6**,**33**,**34*). In contrast, recent reports showed no elevated risk for patients with mild valvulopathy in the ongoing outbreak in the Netherlands (*26**,**27*). Although our univariate analyses showed that nonsurgical cardiac valvulopathy increased the risk for the development of chronic Q fever, this finding was not confirmed in the multivariate analysis. This finding can be explained by the fact that 9/17 (53%) case-patients with nonsurgical valvulopathy also had a history of valvular surgery of one of the other valves. The location and type of valvular defects did not differ significantly between case-patients and controls (Table 2). A possible explanation for the discrepancy with previous observations lies in the fact that our study was conducted 4 years after start of the Q fever epidemic, but chronic Q fever endocarditis in patients with nonsurgical cardiac valvulopathy might become evident later (*6**,**8*). Furthermore, strain-specific differences in clinical signs and symptoms might also be of importance (*26*). Presence of valvulopathy in case-patients and controls could have been missed becausethis was assessed only through review of medical records. However, echocardiography, which was standard care for all patients with suspected cases of chronic Q fever, revealed no additional congenital or bicuspid valve defects, in comparison to assessment of valvulopathy through review of medical records. From other than the above-mentioned defects, it could not be determined by these echocardiograms if these were preexisting or caused by chronic Q fever.

Immunosuppression, although not well defined, has been indicated as a risk factor in former reports, but clear definition and statistical empowerment is lacking (*8*). Although our univariate analysis did show an elevated risk for immunosuppression, especially for patients with proven chronic Q fever cases, this elevated risk was not confirmed in multivariate analysis. Immunocompromised patients may be underrepresented in our study becauseit was conducted in a peripheral hospital setting. Further evaluation of this risk factor should be performed in future studies.

Pregnancy, another formerly reported risk factor, showed an association with the development of chronic Q fever in univariate analysis. However, because there were no pregnant women in the control group and only 3 pregnant women in all case groups, evaluation of pregnancy in multivariate analyses was not possible. A study specifically designed to evaluate associations between pregnancy and Q fever is ongoing in the Netherlands (*35*).

In our opinion, our data were representative for this large Q fever outbreak because they were well documented data and patients were willing to participate. The fact that case-patients and controls were living in the same area increases the comparability of these groups and strengthens the results. However, our study does have potential weaknesses.

First, all controls had an acute episode in 2009, but information about their signs and symptoms was obtained in 2011, introducing possible recall bias. We tried to reduce this bias by requesting additional information from the general practitioners and by reviewing clinical test results and physicians’ reports in the hospital registration systems. Because information bias could also have been introduced by the subjective interpretation of physicians’ reports for case-patients and controls, 2 of our researchers interpreted the results independently.

Serologic follow-up of the controls after the acute Q fever episode lasted only 1 year, which is the normal follow-up period in the Netherlands. However, because chronic Q fever can become manifest years after initial infection, development of chronic Q fever after this follow-up period is still possible (*6**,**22*). Still, 75% of chronic Q fever cases develop within 6 months after primary infection (*22*). Moreover, according to the observed decrease in antibody titers of these patients, progression to chronic Q fever is not likely. In addition, as a consequence of the inclusion of patients with symptomatic acute Q fever as a control group, the results can only be generalized to patients with symptomatic acute Q fever, although the results probably provide an adequate indication of risks factors for patients with mild or asymptomatic primary Q fever.

Notably, almost all controls received antimicrobial drug treatment at time of acute Q fever, in contrast to the case-patients, among which only a minority had symptomatic acute Q fever. Thus, antimicrobial drug treatment might influence the chance of chronic Q fever development, although there is no quantitative evidence that treatment for acute Q fever reduces the chance for chronic Q fever (*4*).

Chronic Q fever cases were selected and classified according to the definitions of the Dutch Q Fever Consensus Group (*20*), which still need confirmation. The definition of probable chronic Q fever contains several patient criteria that we also included as potential risk factors in our study (e.g., valvular disease, vascular prosthesis, aneurysm, and immunosuppressive state). Nevertheless, proven chronic Q fever, for which these criteria are not part of the definition, was also predicted with the identified risk factors in multivariate analysis, thereby confirming the independent risk association of these variables.

Some chronic Q fever cases were identified during screening programs for patients who had valvular surgery, aneurysms, or vascular prostheses. Patients with these risk factors may therefore be overrepresented within our study, although all proven case-patients had symptomatic disease.

Last, the results of this study have to be considered in view of a predominant *C. burnetii* strain that is responsible for the majority of Q fever cases in humans in the Netherlands (*36*). Worldwide, Q fever manifestations differ geographically, which might result from differences in *C. burnetii* strains (*4*).

In conclusion, previous valvular surgery, vascular prosthesis, aneurysms, renal insufficiency, and age were identified as major risk factors for the development of chronic Q fever among persons infected with *C. burnetii*. Because untreated chronic Q fever comes with serious risk for illness and death, awareness is required in people with acute Q fever possessing the identified risk factors. This may require close follow-up or even prophylactic treatment in high-risk groups. Moreover, in case of large Q fever outbreaks, screening is advisable for patients with these identified risk factors.
